# SIREN: suite for intelligent RNAi design and evaluation of nucleotide sequences

**DOI:** 10.1093/bioadv/vbag075

**Published:** 2026-03-16

**Authors:** Pablo Vargas-Mejía, Julio Vega-Arreguín

**Affiliations:** Laboratorio de Ciencias AgroGenómicas, Escuela Nacional de Estudios Superiores Unidad León, UNAM, 37684, León, México; Laboratorio Nacional PlanTECC, Escuela Nacional de Estudios Superiores Unidad León, UNAM, 37684, León, México; Posgrado en Ciencias Biológicas, Universidad Nacional Autónoma de México, Ciudad de Mexico, 04510, México; Laboratorio de Ciencias AgroGenómicas, Escuela Nacional de Estudios Superiores Unidad León, UNAM, 37684, León, México; Laboratorio Nacional PlanTECC, Escuela Nacional de Estudios Superiores Unidad León, UNAM, 37684, León, México

## Abstract

**Motivation:**

RNA interference (RNAi) is a powerful tool for gene silencing across research, therapeutics, and agriculture. However, designing long double-stranded RNAs (dsRNAs) remains challenging because each dsRNA produces many small interfering RNAs (siRNAs), which can collectively introduce substantial off-target effects. Existing tools often lack the ability to account for cumulative off-target interactions, to incorporate thermodynamic modeling, or to accept custom transcriptome inputs, limiting their applicability and accuracy.

**Results:**

Here, we present SIREN, an open-source pipeline designed to streamline RNAi construct design. SIREN integrates siRNA generation, thermodynamically informed off-target prediction, scoring of dsRNA candidates based on cumulative off-target effects, and primer design for *in vitro* synthesis. It accepts user-defined transcriptomes for context-specific analysis and provides adjustable sensitivity settings balancing accuracy and computational demands. Benchmarking across plant, oomycete, and human transcriptomes demonstrates predictable scaling with target length and shows that optional speed modes can reduce runtime while preserving a substantial fraction of high-sensitivity designs and high-risk off-target rankings in many cases. Qualitative validation in *Phytophthora capsici* confirms that SIREN effectively identifies highly specific RNAi constructs with no detectable off-target phenotypes in host plants.

**Availability and implementation:**

SIREN is implemented in Python and available under an open-source license at https://github.com/pablovargasmejia/SIREN.

## 1 Introduction

Since its discovery, RNA interference (RNAi) has become a crucial method for gene silencing, significantly advancing biological research, therapeutics, and agricultural pest control. RNAi primarily relies on double-stranded RNA (dsRNA) molecules processed into small interfering RNAs (siRNAs) that trigger the degradation of complementary mRNA, effectively silencing targeted genes.

Despite RNAi’s efficacy, designing dsRNAs and siRNAs involves significant challenges, particularly off-target effects where unintended genes may be inadvertently silenced, potentially causing undesirable outcomes. Although recent computational and machine-learning methods have enhanced siRNA design by integrating thermodynamic properties, nucleotide positional preferences (e.g. adenine enrichment), and chemical modifications (e.g. 2’-O-methylation), these advances have not been fully extended to comprehensive dsRNA design strategies (Neumeier and Meister 2021, Monopoli *et al.* 2023). Current tools designed for dsRNA, such as si-Fi, rely on homology-based searches (Bowtie) and do not account for the cumulative off-target effects of multiple siRNAs derived from the same dsRNA (Lück *et al.* 2019). Tools like dsCheck consider cumulative effects but do not accept custom transcriptome inputs, restricting their utility to specific model organisms (Naito *et al.* 2005). Other available tools, such as dsRNAEngineer and dsRIP, also lack thermodynamic modeling and do not adequately account for cumulative off-target interactions (Cedden *et al.* 2025, Chen *et al.* 2025).

To address these limitations, we developed SIREN (Suite for Intelligent RNAi Design and Evaluation of Nucleotide Sequences), a bioinformatics pipeline specifically designed to bridge this gap. SIREN systematically generates, evaluates, and ranks RNAi sequences, integrating thermodynamic models and comprehensive cumulative off-target assessment. Importantly, SIREN accepts custom transcriptome inputs, making it broadly applicable beyond model organisms and adaptable to any species of interest. This provides researchers with a powerful tool to enhance precision in gene silencing applications.

## 2 Methods

### 2.1 Overview of the SIREN pipeline

**Figure 1:**
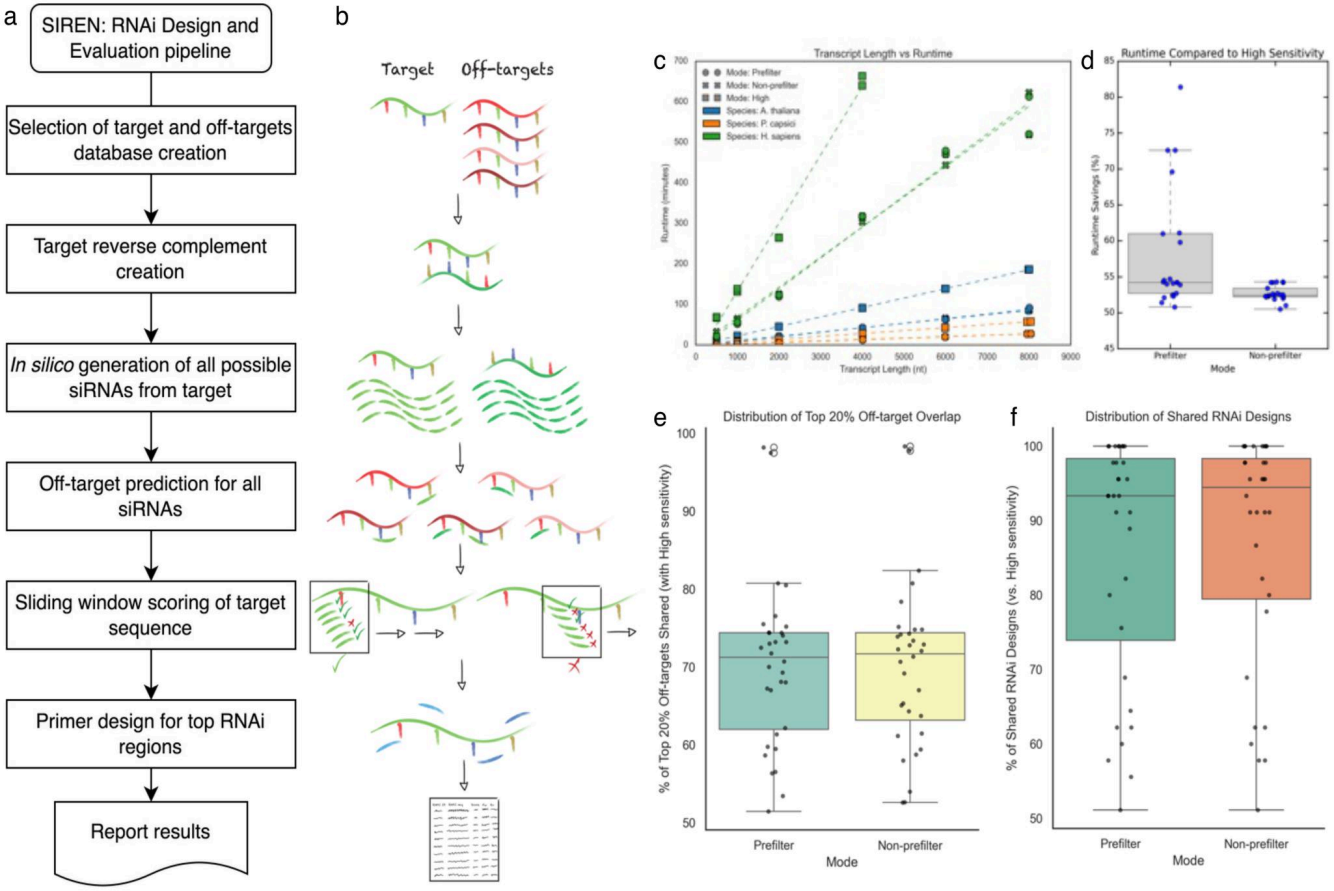
(a) SIREN pipeline overview. (b) SIREN schematic pipeline. (c) SIREN runtime for different run modes, transcriptomes and transcript lengths (48 cores). (d) Runtime of default prefilter and non-prefilter compared to high sensitivity runtime. (e) Percentage of shared RNAi designs between prefilter, non-prefilter and high sensitivity. (f) SIREN prefilter and non-prefilter top 20% off targets shared with high sensitivity.

SIREN is a modular Python workflow for dsRNA design and evaluation. Given a target sequence and a user-defined transcriptome, the pipeline generates candidate siRNAs, evaluates potential off-target interactions, characterizes on-target duplexes, and ranks dsRNA candidates according to cumulative off-target risk. Optional modules include prefiltering, visualization, and primer design (Fig. 1a and b).

### 2.2 Input processing and *in silico* siRNA generation

SIREN initiates by accepting user-provided nucleotide sequences in FASTA format, which may include cDNA, mRNA, or CDS sequences. Alongside these sequences, the user specifies a target gene intended for silencing. First, SIREN generates a reference database of potential off-target sequences derived from the provided input, excluding the target gene to avoid biased predictions. From the forward target sequence, candidate siRNAs are generated by a sliding window (stride set by the selected sensitivity). For each window we also compute its reverse complement (Biopython Seq.reverse_complement) and include both orientations as queries; reverse-complement entries are suffixed “r,” with coordinates reported in the forward orientation. The density of generated siRNAs depends on the user-selected sensitivity option (high or medium). Under high sensitivity (default), SIREN generates overlapping siRNAs at single-nucleotide increments along both strands of the target sequence, maximizing coverage of potential siRNAs and off-target interactions. Medium sensitivity is provided as an optional speed mode that samples siRNAs at 2-nt increments, reducing the number of queries and lowering compute demands at the cost of reduced resolution. All generated siRNA sequences are stored in FASTA format (results_folder/other_files/sirnas.fa).

### 2.3 Prefiltering of candidate off-targets

SIREN includes an optional prefilter (default = on) that reduces the search space before RNAhybrid. In the default windowed seed-density mode, reverse-complement seed k-mers from the target (default length = 9) are scanned across each transcript. A transcript is retained only if at least a user-defined number of seed hits occur within a given window size, thereby enriching for sequences with local clusters of complementarity while discarding unlikely off-targets.

### 2.4 Off-target evaluation

Default parameters (-e -25 -v 0 -u 0 -f 2,7 -p 0.01 -d 0.5,0.1 -m 60000) are selected to reliably identify biologically relevant off-target interactions. To optimize computational efficiency, SIREN utilizes parallel processing, splitting the off-target database into subsets distributed evenly across available CPU threads. The resulting RNAhybrid output is compiled into a single comprehensive file (results_folder/other_files/off_targets_results.txt), from which SIREN produces a summarized three-column table (results_folder/off_targets_summary.tsv). This summary ranks off-target transcripts according to the frequency of siRNA matches, listing each off-target gene, the number of matching siRNAs, and corresponding siRNA identifiers.

### 2.5 Off-target visualization

The pipeline provides an optional graphical representation depicting the positional distribution of siRNAs associated with off-target matches along the target gene. This visualization effectively communicates regions of the target gene generating siRNAs with significant off-target potential.

### 2.6 Scoring, selection, and primer design

Following off-target assessment, SIREN compiles all possible RNAi sequences of user-defined length (default: 300 nt), constructed using a 4-nt step along the target sequence. Each RNAi candidate sequence is evaluated through a scoring algorithm considering two distinct penalties:

1. Off-target siRNA Penalty (−0.1 points):
    A penalty of −0.1 points is applied for each siRNA that fully matches an off-target transcript and is entirely contained within the evaluated RNAi sequence. Each siRNA contributes this penalty only once per RNAi sequence, regardless of how many off-targets it matches, preventing penalty inflation due to multiple off-target hits.
2. Redundant Off-target Hit Penalty (−30 per additional siRNA):
    If multiple distinct siRNAs within a single RNAi candidate target the same off-target transcript, an additional penalty of −30 points is applied for each extra siRNA beyond the first. This ensures strong penalization of RNAi sequences likely to cause extensive unintended silencing of a specific off-target gene.

Scores are calculated efficiently using parallelized computation across multiple CPU threads. From these results, the top 25 candidate RNAi sequences are reported, alongside shorter and longer variants (±50 nt), providing flexible options for experimental implementation. Optimal RNAi candidates undergo primer design via Primer3-py, with options PRIMER_OPT_SIZE: 20, PRIMER_MIN_SIZE: 17, PRIMER_MAX_SIZE: 23, PRIMER_OPT_TM: 60.0, PRIMER_MIN_TM: 55.0, PRIMER_MAX_TM: 63.0, PRIMER_MIN_GC: 40.0, PRIMER_MAX_GC: 60.0, PRIMER_PRODUCT_SIZE_RANGE: [[len(sequence)-20, len(sequence)+20]], PRIMER_NUM_RETURN: 1. Final results, including RNAi sequences, associated scores, and primer pairs, are provided in the file results_folder/rna_sequences_with_scores.tsv (Fig. 1a and b). Scoring system validation scripts, inputs, and reports for the scoring-weight exploration and the experimental *Drosophila* dsRNA benchmark are available at https://github.com/pablovargasmejia/SIREN/tree/main/scoring_validation_dsRNA_data and https://github.com/pablovargasmejia/SIREN/tree/main/scoring_validation/.

### 2.7 Benchmark and testing

Benchmarking was designed to characterize runtime scaling and practical output concordance across operating systems and transcriptome sizes, rather than to provide inferential statistical comparisons. We evaluated three representative reference transcriptomes: *Arabidopsis thaliana* (TAIR10 from arabidopsis.org), *Phytophthora capsici* (LT1534 v11.0 from MycoCosm), and *Homo sapiens* (GRCh38 from ENSEMBL). For each reference set, we sampled twenty target transcripts spanning a range of lengths (∼0.5–8 kb) to probe scaling behavior under typical use cases. Each target was run using the same pipeline stages and RNAhybrid settings, varying only the sensitivity mode (high versus medium) and whether the optional prefilter was enabled. All benchmarks and concordance analyses reported in this version were generated with the current implementation and current sensitivity definitions. The pipeline was tested on the Ohio Supercomputer Center (OSC) using 48 CPUs. All benchmark datasets, scripts, and environment specifications required to reproduce the analyses are publicly available in the GitHub repository under the /benchmark directory.

## 3 Results

### 3.1 Performance

SIREN was evaluated across multiple operating systems, including macOS Sequoia, Ubuntu, and Red Hat Enterprise Linux (RHEL). Benchmarks were performed on an RHEL 9.4 cluster comprising 48 cores. Three transcriptome databases of increasing complexity were tested: *Phytophthora capsici* (20.4 Mb; 19 805 transcripts; mean transcript length 1028.2 bp; SD 890 bp), *Arabidopsis thaliana* (64.9 Mb; 41 671 transcripts; mean transcript length 1556.6 bp; SD 1105.1 bp), and *Homo sapiens* (395.1 Mb; 207 133 transcripts; mean transcript length 1907.6 bp; SD 2170.6 bp).

At high sensitivity, runtime scaled linearly with transcript length for all three organisms, but with organism-specific slopes that reflect database complexity: *P. capsici* 7.21 min kb^−1^, *A. thaliana* 23.37 min kb^−1^, and *H. sapiens* 167.86 min kb^−1^ (Fig. 1c). The windowed prefilter reduced wall-time by a median of 54%–57% across species (≈2.17–2.34× faster than high sensitivity). Non-prefilter (medium sensitivity) mode also cut times by ≈52% overall (≈2.09–2.18× faster) (Fig. 1c and d). Per-nucleotide rates showed the same pattern: medians fell from 0.421 to 0.194 s/nt (*P. capsici*), 1.378 to 0.631 s/nt (*Arabidopsis*), and 8.099 to 3.442 s/nt (human) with prefiltering. Overall, SIREN exhibits predictable linear scaling with length, while filtering strategies deliver ∼2× speedups without compromising high-sensitivity settings.

More than 94% of total runtime was consistently spent on the off-target search step, which relies on RNAhybrid. This indicates that computational cost in the current implementation is dominated by thermodynamic duplex evaluation rather than upstream orchestration steps. Although homology-based aligners such as Bowtie or BLAST are computationally faster, they do not explicitly model hybridization energetics. In contrast, RNAhybrid evaluates duplex formation using minimum free energy calculations, providing a thermodynamically informed framework for off-target prediction (Chen *et al.* 2019, Riolo *et al.* 2021). Accordingly, off-target evaluation remains the main target for future optimization in SIREN.

### 3.2 Sensitivity levels

SIREN’s sensitivity setting controls the density of siRNAs generated along the target. High sensitivity (default) generates siRNAs at 1-nt steps on both strands, yielding up to 2 × (target length − siRNA length + 1) siRNAs. Medium sensitivity is an optional speed mode intended to reduce computational effort on limited hardware; in the current version it uses a 2-nt stride, reducing the siRNA set by ∼50%.

Benchmarking was performed on targets drawn from three transcriptomes (*Phytophthora capsici* MycoCosm cDNA, *Arabidopsis thaliana* TAIR10 cDNA, and human Ensembl cDNA). For each target, runs were performed under matched conditions across modes (medium non-prefilter, medium + prefilter, and high). At medium sensitivity, exact recovery of high-sensitivity RNAi designs ranged from 52.6% to 100%, with medians of 93.3% (non-prefilter) and 94.4% (prefilter) (Fig. 1e). High sensitivity was reported between 1063 and 100 560 off-targets (median ≈ 10 370). Medium sensitivity produced similar totals without prefiltering (median ≈ 10 264) and a stronger reduction with prefiltering (median ≈ 9863; ∼13% reduction) (Fig. 1e).

To focus on the highest-risk off-targets, we defined the top 20% redundant off-targets as those in the upper quintile of “siRNA number” (transcripts targeted by many distinct siRNAs). Medium sensitivity preserved substantial concordance with high sensitivity: the median overlap of this high-risk subset was 71.7% (non-prefilter) and 70.2% (prefilter), with Spearman correlations of redundant off-target counts showing medians of 0.91 (non-prefilter) and 0.92 (prefilter), ranging from 0.84 to 0.98 (Fig. 1f). Overall, medium sensitivity retains most high-priority off-target structure while reducing computational demand, whereas high sensitivity remains the default for maximal coverage.

### 3.3 Real RNAi tests

SIREN was experimentally validated by silencing multiple *P. capsici* genes through application of dsRNA onto pathogen and host tissues (leaves and stems). dsRNAs were designed using SIREN against a combined transcriptome database of the phytopathogen *P. capsici* and four different plant hosts: tomato, chili pepper, cucumber, and melon. Following synthesis (cDNA amplification, T7 promoter fusion PCR, and T7-based in vitro transcription), none of the designed dsRNAs caused detectable off-target phenotypes in host plants, confirming practical effectiveness (Vargas-Mejía *et al.* 2026).

### 3.4 Scoring system

To provide experimental support that SIREN’s ranking behavior avoids empirically off-target–prone dsRNA regions, we benchmarked SIREN using a published *Drosophila melanogaster* cell-based dataset reporting long dsRNAs with documented off-target effects (Kulkarni *et al.* 2006). For each of several genes, this study tested three long-dsRNA amplicons (Amp1 to Amp3), where Amp1 was associated with off-target effects while Amp2 and Amp3 were not. Using the FlyBase transcriptome as the off-target database, we ran SIREN on the corresponding FlyBase gene identifiers, matching the RNAi length in each run to the exact length of the experimental amplicon, and we evaluated whether SIREN’s top-ranked designs overlapped the mapped Amp1 region versus Amp2 or Amp3 on the exact target sequence used by SIREN in that run. Across the completed runs (excluding one still-running large job), SIREN’s number 1 candidate did not overlap Amp1 under a conservative overlap threshold of 50 bp, and whenever the top-ranked candidate overlapped a published amplicon, it overlapped Amp2 or Amp3 rather than Amp1. This experimentally anchored result supports that SIREN’s scoring and ranking preferentially avoid regions associated with observed off-target effects, and the full workflow (inputs, evaluation scripts, and reports) is publicly available at https://github.com/pablovargasmejia/SIREN/.

## 4 Conclusions

SIREN facilitates the design and selection of highly specific RNAi constructs by explicitly modeling cumulative off-target effects across all siRNAs derived from a dsRNA candidate. Benchmarking demonstrates predictable scaling with target length, with runtime dominated by the RNAhybrid off-target search. High sensitivity is the default and recommended configuration to maximize coverage of potential siRNAs and off-target interactions. Medium sensitivity is provided as an optional speed mode to reduce computational effort on constrained hardware, with the expectation that outputs may differ from high sensitivity depending on target sequence properties and transcriptome complexity. Experimental validation underscores SIREN’s practical utility, ensuring reliable RNAi design with minimal unintended off-target outcomes. In practice, SIREN is well suited for non-model species without curated resources, for pathogen-host interaction studies requiring custom transcriptomes, and for agricultural applications where off-target phenotypes must be minimized.

The current version has some limitations: runtime is dominated by RNAhybrid-based off-target searches, and biological filters such as small RNA biogenesis preferences, structural accessibility, or cross-kingdom dynamics are not yet integrated. Future development will therefore focus on accelerating off-target evaluation (e.g. through batched or approximate search), incorporating additional biological context, and expanding support for high-throughput validation pipelines. These steps will strengthen SIREN as a versatile tool across research, therapeutic, and agricultural settings.

## Data Availability

The data underlying this article are available in GitHub and PyPi: https://github.com/pablovargasmejia/SIREN, https://pypi.org/project/siren-rnai/.
